# DNA-Methylation Profiling of Fetal Tissues Reveals Marked Epigenetic Differences between Chorionic and Amniotic Samples

**DOI:** 10.1371/journal.pone.0039014

**Published:** 2012-06-19

**Authors:** Christel Eckmann-Scholz, Susanne Bens, Julia Kolarova, Sina Schneppenheim, Almuth Caliebe, Simone Heidemann, Constantin von Kaisenberg, Monika Kautza, Walter Jonat, Reiner Siebert, Ole Ammerpohl

**Affiliations:** 1 Department of Gynecology & Obstetrics, Christian-Albrechts-University Kiel & University Hospital Schleswig-Holstein, Kiel, Germany; 2 Institute of Human Genetics, Christian-Albrechts-University Kiel & University Hospital Schleswig-Holstein, Kiel, Germany; 3 Department of Obstetrics, Gynecology and Reproductive Medicine, Hannover Medical School, Hannover, Germany; 4 Praxis of Human Genetics, Kiel, Germany; RWTH Aachen University Medical School, Germany

## Abstract

Epigenetic mechanisms including DNA methylation are supposed to play a key role in fetal development. Here we have investigated fetal DNA-methylation levels of 27,578 CpG loci in 47 chorionic villi (CVS) and 16 amniotic cell (AC) samples. Methylation levels differed significantly between karyotypically normal AC and CVS for 2,014 genes. AC showed more extreme DNA-methylation levels of these genes than CVS and the differentially methylated genes are significantly enriched for processes characteristic for the different cell types sampled. Furthermore, we identified 404 genes differentially methylated in CVS with trisomy 21. These genes were significantly enriched for high CG dinucleotid (CpG) content and developmental processes associated with Down syndrome. Our study points to major tissue-specific differences of fetal DNA-methylation and gives rise to the hypothesis that part of the Down syndrome phenotype is epigenetically programmed in the first trimester of pregnancy.

## Introduction

The epigenetic composition of the fetus has gained much attention in the recent past. In particular, the patterns of DNA methylation and their changes during normal and pathologic fetal development are subject of studies in various fields of research. These include reproduction failure, effects of assisted reproduction, disturbances of the maternal-fetal interface, disorders of development, syndromes caused by errors in imprinting, fetal programming of behavior or disease or the impact of maternal factors on fetal development [1]–[4].

Compared to its sequence the methylation of DNA shows a much higher plasticity both intra- and interindividually. DNA methylation patterns differ between tissues and epigenetic modifications are supposed to be major determinants of the cell type-specific gene expression program and, thus, to imprint the cellular protein expression, function and destiny [5], [6]. Consequently, DNA methylation patterns can be influenced by internal and external factors allowing cellular differentiation and response to exogenic stimuli. The latter is directly linked to the interindividual differences in DNA methylation which have been shown to increase with ageing by twin studies [7]–[9]. Considering this epigenetic plasticity it is intriguing to speculate that differences in DNA methylation patterns could explain (part of the) phenotypic differences observed between individuals carrying the same genetic change. E.g., despite carrying a third chromosome 21 only 40–60% of individuals with Down syndrome are affected by congenital heart defects, mostly an atrial (ventricular) septal defect [10]–[12].

This figure - besides through the effect of modifier genes - could well be explained by a secondary epigenetic mark influencing cardiac development established or erased directly or indirectly through the effect of a gene on chromosome 21.

DNA methylation patterns can also be of relevance for diagnostics. In this regard, a series of locus-specific and genome-wide DNA methylation profiling studies has been performed in the recent past in order to detect methylation patterns differentiating fetuses with chromosomal aberrations from those with normal karyotypes. This led to the development of novel approaches for noninvasive prenatal diagnosis of e.g. trisomy 21 by analysis of fetal-specific DNA methylation in maternal blood [13].

It is remarkable, that description of fetal trisomic and non-trisomic fetal DNA methylation patterns, respectively, has so far been almost exclusively based on the analysis of a limited number of placental DNA both from first and third trimester pregnancies [14]–[18]. Nevertheless, it is well known from prenatal chromosomal testing that placental tissue e.g. derived from chorionic villi sampling (CVS) is a peculiar fetal tissue to study because of the mixture of ectodermal and mesodermal cells, recurrent mosaicism and the contamination of fetal tissue by maternal cells. In contrast to those placental cells which are more distantly related to the fetus, cells obtained by amniocentesis (AC) which are epithelial cells derived from the epiblast of the inner cell mass are supposed to reflect closely the true composition of the fetus [19].

In order to characterize and compare DNA methylation patterns between amniotic and chorionic tissue samples we performed an array-based methylation profiling study in an extensive series of 63 fetal tissues derived from routine prenatal AC and CVS sampling. Through analysis of CVS samples from fetuses with trisomy 21 we unravel potential novel markers for non-invasive prenatal testing. Finally, by describing the molecular features shared by the genes differentially methylated between the different tissues as well as the CVS samples with and without trisomy 21 we provide initial insights into the biological processes underlying epigenetic patterns in normal fetal differentiation and development as well as potentially in the manifestation of the typical phenotype of Down syndrome.

## Materials and Methods

### Study Population

The study was performed on 63 samples from 61 different women left over from routine prenatal cytogenetic testing. This included 47 chorionic villi samples (CVS) and 16 samples derived from amniocentesis (AC). CVS and AC sampling was performed with written informed consent of the patients according to standard methods. The main indications for invasive prenatal testing were advanced maternal age as well as abnormal ultrasound findings such as elevated nuchal translucency (NT) or abnormal first trimester screening. Clinical details on the pregnancies as well as pregnancy outcome are summarized in Table S1. For control purposes peripheral blood samples from normal female and male individuals were included. The study was approved by the Ethics Committee of the Medical Faculty of the Christian-Albrechts-University Kiel (IRB No: D447/09, D402/09).

### DNA Extraction

DNA was extracted from native CVS tissue left over after setting up direct preparations and long-term cultures for cytogenetic analyses as well as from long-term cultured AC cells. Samples were cryopreserved until DNA extraction. DNA extraction was performed centrally by a single person to widely exclude batch effects applying the High Pure PCR Template Preparation Kit (Roche Diagnostics, Mannheim, Germany).

### DNA Methylation Analysis

DNA methylation analysis using the HumanMethylation27k BeadChip (Illumina Inc., San Diego, CA, USA) was performed using a custom service provided by AROS Applied Biotechnology AS (Aarhus, DK). The HumanMethylation27 BeadChip was developed to assay 27,578 CpG sites selected from more than 14,000 genes in parallel [20]. Two samples were analyzed in duplicates using different batches of the HumanMethylation27 BeadChip. Array data are available in a MIAMI compliant format from GeneOmnibus (GSE35327).

### Processing of Data and Bioinformatic Analyses

Raw hybridisation signals were processed using GenomeStudio software (ver. 2011.1; Illumina Inc.) applying the default settings. Further analyses were performed using the R-package [21] and RStudio (ver. 0.94.102) using raw data obtained from the GenomeStudio analysis. Color balance adjustment and data normalization (*simple scaling normalization*) were done using the lumi package for R [22]–[24]. CpG loci with detection p-values<0.001 in at least one of the samples analyzed were excluded from further analyses. Finally, 22234 CpG loci in 63 samples entered analysis. Data obtained from samples analyzed in duplicates were averaged. Subsequent principal component (PCA) and hierarchical cluster analyses were performed using Qlucore’s Omics Explorer 2.1 (Version 2.1(25); Qlucore, Lund, Sweden). Genes were considered being differentially methylated between two data sets if the false discovery rate (FDR) was below q<0.01 (t-test). The R-package was used to perform Pearson's product-moment correlation.

### Bioinformatic Characterisation of Groups of Differentially Methylated Genes

To analyze whether promoter regions of differentially methylated genes showed different CpG compositions, we used a previously described classification into promoters with high (HCP), intermediate (ICP) and low (LCP) CpG content [25], [26]. Proportions of PRC2 target genes and promoter classes in the groups of genes differentially methylated and all genes present on the HumanMethylation27 Bead Chip were compared using the chi-square test (two-sided; GraphPad Prism, ver. 4.02). A genome-wide mapping of PRC2 genes in embryonic stem cells is available as supplemental material in the study by Lee et al. [27]. Imprinted genes were identified from publicly available databases (http://igc.otago.ac.nz/home.html26 and http://www.geneimprint.com/site/genes-by-species27) and a previously published review [28]. Gene ontology analysis has been performed using GOrilla [29] with a list of genes present on the BeadChip acting as background list.

### Bisulfite Pyrosequencing of KIAA0575, ECEL1, SOX11 and STG4

Bisulfite conversion of genomic DNA was performed using the EpiTect Bisulfite Conversion Kit (Qiagen, Hilden, Germany) according to the manufacture’s protocol. Bisulfite Pyrosequencing was performed according to previously published protocols [30]. Primer sequences and precise PCR conditions are available from the authors on request. DNA-methylation of *KIAA0575*, *ECEL1*, *SOX11* and *STG4* has been analyzed in this study.

#### GeneScan analysis of the *AMELX/AMELY* locus

To determine the content of fetal tissue in CVS samples obtained from male fetuses we performed a PCR based GeneScan analysis on the *AME*L gene locus using primers yielding different sized PCR fragments from the X-chromosomal (*AMELX*, Xp22.2) and Y-chromosomal (*AMELY*, Yp11.2) copy of the gene. PCR fragments were analyzed using an ABI 3100 (Applied Biosystems, Carlsbad, CA). DNAs from peripheral blood of normal males (n = 6), normal females (n = 5) as well as mixtures of normal male and female DNA (ratio: 1∶1, n = 5) acted as control. The ratios of the peak area of X- and Y-chromosomal specific peaks were calculated. Using the controls as standard values in a regression analysis, the content of fetal tissue in CVS samples has been determined in CVS samples from male fetuses.

## Results

Applying the “Infinium HumanMethylation27 BeadChip” we investigated 27,578 CpG sites selected from more than 14,000 genes in a series of 63 fetal tissues left over from routine prenatal genetic testing. In particular, we investigated 47 chorionic villi samples (CVS) and 16 samples derived from amniocentesis (AC). Besides samples from fetuses with normal male or female karyotype these included six cases with trisomy 18 (5 CVS, 1 AC) and three cases with trisomy 21 (all CVS). One CVS sample was derived from a fetus with a gonosomal chromosome pattern typical for Turner Syndrome. Two samples (1 AC, 1 CVS with trisomy 21) were obtained from pregnancies induced by intracytoplasmatic sperm injection (ICSI). None of the chromosomal aberrations was mosaic. Clinical details are summarized in Table S1.

The reproducibility and validity of the applied array platform for determination of DNA-methylation levels has been extensively demonstrated by us in previous studies using different types of samples and comparison to alternative techniques like methylation specific PCR, bisulfite pyrosequencing or bisulfite sequencing [31]–[33]. In addition, we here investigated the reproducibility by analyzing two pairs of CVS samples in duplicate (CV21 and 19; CV26 and 10; Table S1) using two different batches of the array. This comparison yielded Pearson correlation coefficients of 0.96 and 0.97, respectively, indicating good reproducibility also in the fetal samples under study (Figure S1). Moreover, to test the validity of the DNA methylation values derived from the array we here examined the status of 6 CpG loci in 3 randomly selected AC und 7 CVS samples by bisulfite pyrosequencing (Figure S2).

**Figure 1:**
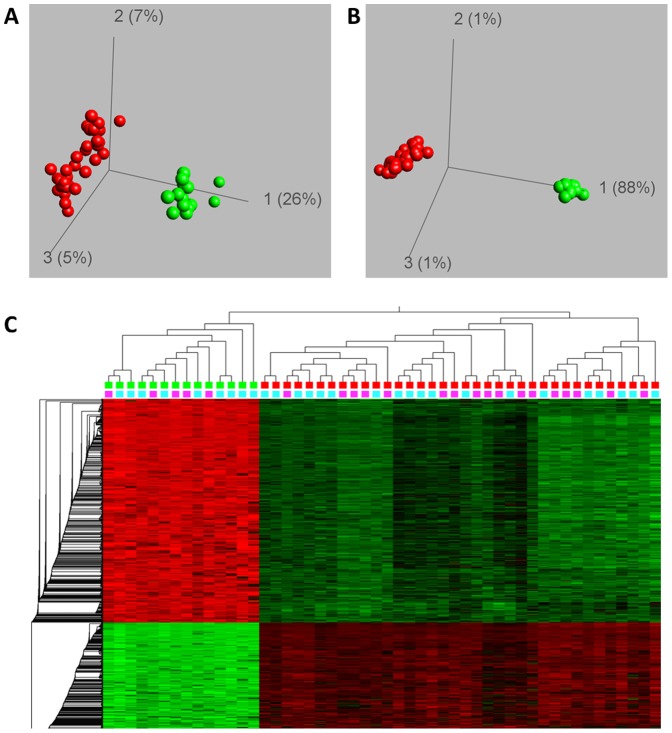
DNA methylation differs between AC and CVS samples. (A) Principal component analysis (PCA) of 22,234 CpG loci which passed quality tests and entered the analysis. Unsupervised analysis separated AC (green spheres) from CVS samples (red spheres). (B) Differential methylation analysis to separate AC from CVS samples (t-test, FDR<1×10^−15^). (C) Hierarchic cluster analysis of the 2418 CpG loci shown in (B). The upper panel on top of the heatmap indicates the sample type (green: AC, red:CVS) while the second panel indicates the fetuses’ sex (pink: female, cyan: male). Green color in the heatmap indicates low DNA-methylation, black intermediate and red high DNA-methylation values. Samples with known chromosomal aberrations and samples from ICSI were excluded. AC and CVS differ significantly in their DNA methylation pattern.

Having established the technical validity of the array we aimed in a first step at comparing the DNA-methylation between the amniotic cells and the chorionic samples with a regular karyotype. To this end, we excluded all samples with a chromosomal aberration. Moreover, we excluded the samples of pregnancies derived after ICSI in order to avoid putative effects of in vitro fertilization on the epigenetic pattern. Already an unsupervised principal component analysis (PCA) of the complete data set from the remaining 50 samples clearly separated AC from CVS samples (Figure 1a). This indicates that the methylation patterns of the samples investigated are much stronger associated with the origin of the fetal tissue than other factors like sex of the fetus despite the X-chromosome undergoing DNA-methylation in females is being included in the analysis. A supervised comparison identified 2418 CpG loci corresponding to 2014 individual genes to be significantly (FDR<1×10^−15^, t-test) differentially methylated between AC and CVS (Figures 1b and 1c). Like in the unsupervised analysis the samples clustered independently from the sex of the fetus (Figure 1c). We conclude that there are major differences in the DNA-methylation patterns between the fetal tissues obtained by routine invasive prenatal testing.

**Figure 2:**
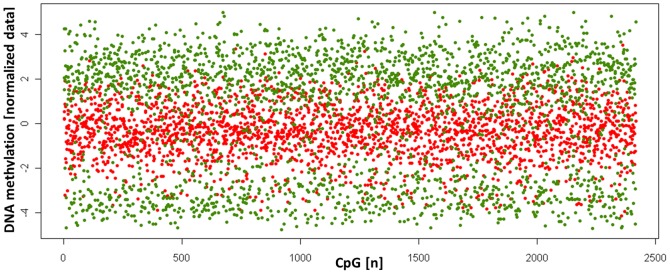
CpG loci differentially methylated between AC and CVS samples preferentially show an intermediate methylation level in CVS. Scatter plot of the means of DNA methylation values of all 2418 CpG loci differentially methylated between samples obtained by amniocentesis (AC, green circle) and chorionic villi biopsy (CVS, red circle) samples (FDR<1×10^−15^). For all loci the means have been calculated in AC and CVS separately and subsequently plotted. Most CpG loci are characterized by either a high or a low DNA methylation value in AC but an intermediate DNA methylation value in CVS.

Interestingly, inspection of the heatmap (Figure 1c) suggested that the methylation values of the genes differentially methylated between amniotic and chorionic samples are more extreme in AC than those in CVS. To further investigate this observation, we plotted the means of the normalized methylation values in AC and CVS of all differentially methylated 2418 CpG loci. As shown in Figure 2, these loci were preferentially (2054 of 2418 CpG loci) intermediately methylated in CVS but either lowly or highly DNA-methylated in AC samples. In contrast, genes not differentially methylated between CVS and AC showed a similar DNA methylation distribution pattern in both sample types (Figure S3). We conclude that the genes differentially methylated between AC and CVS are predominately characterized by more extreme DNA-methylation levels in the former and more intermediate levels in the latter samples.

**Figure 3:**
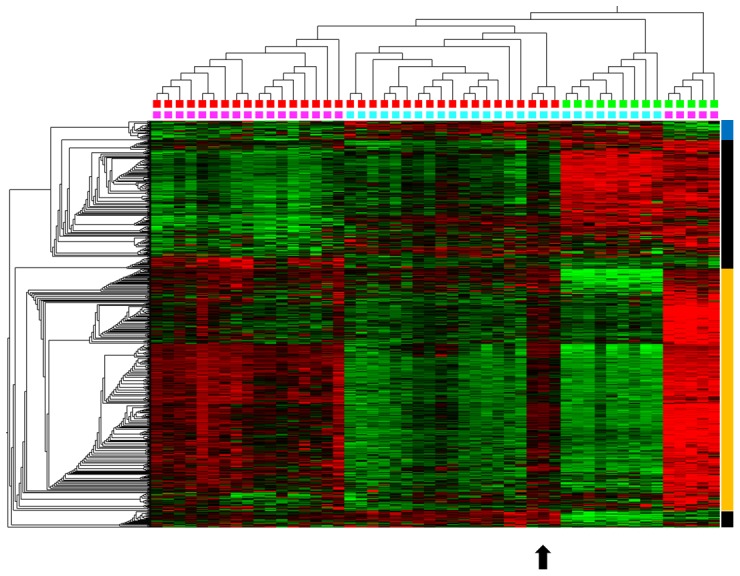
Hierarchic cluster analysis of DNA methylation values of 767 X-chromosomal CpG loci present on the array. The upper panel on top of the heatmap indicates the sample type (green: AC, red: CVS) while the second panel indicates the fetuses’ sex (pink: female, cyan: male). Samples with known chromosomal aberrations and samples from ICSI were excluded. Three CVS samples from male fetuses show a DNA methylation pattern similar to female samples (arrow). A blue bar at the right site indicates genes methylated specifically in male fetuses, an orange bar genes methylated in female samples and a black bar genes with tissue- specific DNA methylation.

The more intermediate methylation levels of the genes differentially methylated between amniotic and chorionic samples could be due to the fact that tissue sampled by CVS is a mixture of various embryonic and also maternal cell compartments. In contrast, cells derived from AC usually lack maternal contamination if the sample is not contaminated by blood. The potential differences in the contribution of maternal cells between AC and CVS prompted us to investigate the correlation of the DNA-methylation patterns with fetal sex. Unsupervised analysis of the 50 samples already suggested that fetal sex was less visible in the methylation pattern of CVS than in AC. To corroborate this observation we focused on the X-chromosomal genes as many (but not all) of those are being methylated in 50% of the X-chromosomes of females due to X-inactivation not present in normal males. In an unsupervised approach both AC and CVS samples separated according to fetal sex based on the methylation levels of the X-chromosomal genes. Nevertheless, three CVS samples from male fetuses clustered separately from the other CVS samples of male fetuses (Figure 3, arrow). These three samples showed a mixture of male and female X-chromosomal methylation patterns likely due to maternal cells contaminating the CVS sample. We conclude that varying maternal contribution affects the DNA methylation pattern of CVS samples. This is further supported by a GeneScan analysis of the *AMELX*/*AMELY* locus in CVS samples derived from male fetuses revealing a detectable maternal contamination of between 0% and 29% (median 17%).

Next we wondered whether the 2014 genes differentially methylated between amniotic and chorionic samples share certain biological features. Several studies demonstrated that genes with promoters either containing a high CpG content (HCP) or a low CpG content (LCP) show different DNA methylation properties [31]–[33]. The 628 genes found hypermethylated in CVS compared to AC were significantly enriched for polycomb repressor complex 2 (PRC2) target genes in embryonic stem cells (p<0.0001; OR = 3.88, RR = 3.51) and imprinted genes (p<0.0001, OR = 2.69, RR = 2.50), while LCP genes were depleted (p = 0.0213, OR = 0.77, RR = 0.78). In contrast, the 1406 genes hypermethylated in AC in comparison to CVS were significantly enriched for genes with LCPs (p<0.0001, OR = 4.39, RR = 3.62) but depleted for HCP genes (p<0.0001, RR = 0.29, OR = 0.26) and PRC2 target genes in embryonic stem cells (p<0.0001, RR = 0.35, OR = 0.33).

We further investigated whether genes differentially methylated between AC and CVS were enriched for certain biological functions by performing a gene ontology analysis. This approach revealed that the 2014 genes differentially methylated between AC and CVS were significantly enriched for genes involved in various regulatory and developmental processes including e.g. regulation of adenylate cyclase activity which might point e.g. to hormonal or neuronal signaling (Table S2). In addition, the physiologic properties of the cell compartments analyzed are reflected by significant enrichment of the differentially methylated genes for processes “(cellular) response to nitric oxide” (5.3–6.3fold; containing predominately genes encoding surfactant proteins), “keratinization” (2.8fold) or “female pregnancy” (2.4fold). A detailed list of loci aberrantly methylated in CVS samples with trisomy 21 and trisomy 18 along with the corresponding DNA methylation values of the respective loci in normal peripheral blood is available from the supplement (Table S4). Applying a threshold of delta.beta>0.2 we identified 89 loci with differential methylation between CVS with trisomy 21 and both normal CVS as well as peripheral blood samples from normal females. All of these loci were hypermethylated in trisomy 21 samples.

One single locus (CACNA1H, cg25760229) was differentially methylated between CVS with trisomy 18 and both normal CVS and peripheral blood samples from normal females (hypermethylated in trisomy 18 samples).

Finally, we wondered whether maternal factors could be associated with methylation differences in CVS and AC samples with normal karyotype. Due to the tissue differences shown above we treated both sample sources differently. By PCA we failed to detect any significant effect of maternal age, BMI or smoking status on the methylation pattern in AC and CVS, respectively (data not shown). Probably, the number of samples under investigation was too low to get a conclusive significant result.

**Figure 4:**
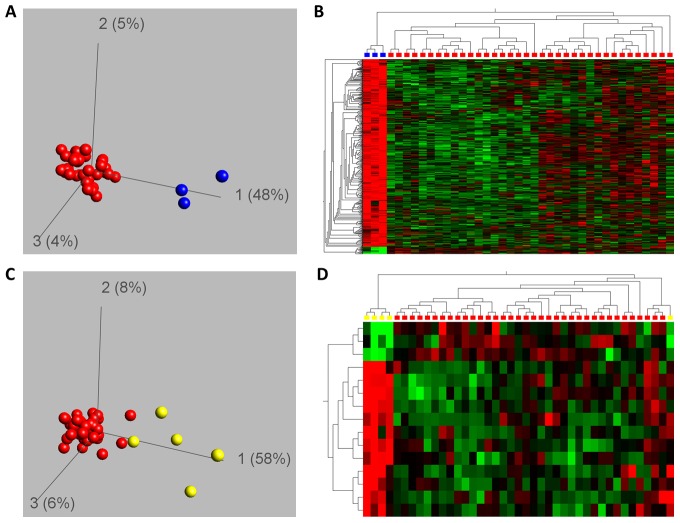
DNA methylation patterns in CVS samples with chromosomal imbalances. PCA (A) and Hierarchic cluster analysis (B) of DNA methylation values of 464 CpG loci differentially methylated between chorionic villi biopsy samples without chromosomal imbalances (red spheres or red squares, respectively) and samples with trisomy 21 (47,XY+21; blue spheres or blue squares, respectively) (FDR<0.01). While in cases with trisomy 21 numerous epigenetic alterations could be identified, in cases with trisomy 18 (47,XX+21/47,XY+21, yellow spheres or yellow squares) only 15 differentially methylated loci have been found (C and D). Green color in the heatmap indicates low DNA-methylation, black intermediate and red high DNA-methylation values.

Having determined the methylation patterns in AC and CVS samples with normal karyotypes we aimed in the second part of the analyses at investigating whether fetal trisomies were associated with an altered DNA-methylation pattern. We limited the bioinformatic analyses to the CVS samples as the number of fetuses with a trisomy in the AC population was too low. In the three CVS samples with a fetal trisomy 21 (including one conceived by ICSI) a total of 464 loci (Figure 4; q<0.01) corresponding to 404 genes were differentially methylated as compared to CVS samples with a normal karyotype. Remarkably, the vast majority (387/404) of the genes was hypermethylated in the trisomic samples and only 17 hypomethylated. The 387 hypermethylated genes in trisomy 21 were significantly enriched for genes with HCP content (p = 0.0244, OR = 1.27, RR = 1.28), known PRC2 target genes in embryonic stem cells (p<0.0001, OR = 4.37, RR = 4.07) and (known or suggested) imprinted genes (p<0.0001, OR = 3.23, RR = 3.03), while genes with LCP were significantly depleted (p = 0.0002, OR = 0.55, RR = 0.56). In contrast the 17 hypomethylated genes were significantly enriched only for genes with HCP content (p = 0.0146, OR = 5.93, RR = 5.92). Gene ontology analysis of all 404 aberrantly methylated genes in cases with trisomy 21 showed a significant enrichment of several processes in development, embryogenesis and signaling which could be associated with the typical phenotype of Down syndrome, including “neuron fate determination” (15.2fold), “meso/metanephros development” (9.4fold, 5.9fold), “heart morphogenesis” (5.5fold), “eating/feeding behavior” (11.9fold, 6.2fold) or “embryonic digestive tract morphogenesis” (9.5fold) (Table S3).

In contrast to the samples with trisomy 21, an analysis comparing the five CVS samples with trisomy 18 to the normal controls identified only 15 significantly differentially methylated loci, corresponding to 14 genes (Figures 4c and 4d). No significant enrichment of any properties of the genes was detected.

Finally, we wondered whether the single sample of AC with trisomy 18, the CVS sample with a gonosomal chromosome pattern typical for Turner Syndrome and the AC samples derived from a pregnancy after ICSI showed peculiar patterns of methylation. Obviously, bioinformatic analysis of single samples is meaningless. Nevertheless, displaying the methylation pattern of the X-chromosomal genes in the Turner sample as compared to the mean values of the normal male and female CVS samples showed lack of both, the typical female X-inactivation pattern and of the male methylation pattern (Figure S4). Finally, displaying the two ICSI samples as compared to the normal AC and CVS samples with regard to the genes differentially methylated between both tissues showed a remarkable loss of methylation in the ICSI derived AC sample (Figure S5). This might point to changes in the DNA methylation detectable in AC samples derived from ICSI patients but warrants further investigation.

## Discussion

In the present study we performed array-based methylation profiling on an extensive series of fetal samples with normal and aberrant karyotypes obtained by routine prenatal CVS and AC sampling. Obviously, relying on left-over samples from routine prenatal testing implements a certain selection bias as with few exceptions only fetuses at risk for chromosomal aberrations and/or with abnormal ultrasound findings or maternal serum screenings will be sampled due to ethical considerations. Thus, the present series was enriched for pregnancies with normal karyotype but abnormal ultrasound (n = 12), aberrant first trimester screen (n = 23) or advanced maternal age (n = 41) (multiple answers were permitted). Moreover, samples with known chromosomal aberrations were included on purpose. Thus, the analyses provided here reflect the findings of an “at-risk” cohort rather than of the normal distribution of pregnancies.

Moreover, it needs to be taken into account that whereas native (cryopreserved) CVS samples were investigated without prior culturing the AC cells had to be cultured to obtain sufficient amounts of DNA for the analyses. In this regard it needs to be considered that amplification of unmodified DNA prepared from uncultured AC cells would not have been suitable for the methylation arrays applied as by the amplification process the DNA methylation marks get lost. In turn, amplification of bisulfite treated DNA might have introduced experimental biases due to selective amplification of methylated or unmethylated DNA. The minute DNA amounts left over after routine diagnostics from uncultured amniotic cells might also be the reason why hitherto genome-wide methylation profiling studies focused on CVS or placental samples. Obviously, culturing cells harbors the risk of changing the DNA methylation pattern. Nevertheless, based on our own and others reports on lymphoblastoid cell lines as compared to normal lymphocytes we think that the culture-induced methylation changes are minor as compared to the tissue-specific differences which were in the focus of the present study [31]. To further evaluate this assumption we determined the predicted cumulative population doublings, predicted passages and the predicted time in culture using the tool available at http://www.molcell.rwth-aachen.de/dms. Based on the DNA methylation values of seven CpG loci present on the HumanMethylation27k Bead Chip Koch et al. developed an algorithm to investigate these parameters from in vitro cultured cells [34]. As calculated by this tool, the cumulative population doublings ranged from 1 to 26 (median = 9), the predicted passages from −2 to 12 (median = 2) and the predicted time in culture from 18 to 64 (median = 7.5) in AC samples. All these values were lower than those calculated from uncultured CVS cells supporting our assumption that the methylome of the in vitro cultured AC samples might not be heavily altered in culture.

Even taking into account potential sampling and culturing artifacts the differences in the methylation patterns between CVS and AC samples with normal karyotype were striking. Investigating nearly 800 selected genes using the Illumina GoldenGate DNA Methylation Cancer Panel I array, Yuen et al. [35] recently identified 195 sites (23%) to be differentially methylated between various fetal studies. Here 2418 CpG loci corresponding to 2014 individual genes, i.e. almost 11% of all CpGs on the array, showed a differential methylation between the two tissues studied. This strong difference is most likely attributed to the fact that CVS samples derive from chorionic ectoderm (trophoblast) and extra-embryonic mesoderm and blood whereas cells sampled by AC are all of embryonic ectodermal origin including epithelial cells from the embryonic surfaces and from the amnion [19]. Indeed, the gene ontology analyses support this view showing on the one hand enrichment of genes involved in more fetal ectodermal or epithelial functions like keratinization or response to nitric oxide (mediated by surfactant proteins) and on the other hand more trophoblastic and extra-embryonic functions including many pregnancy associated hormones and proteins (see Table S2).

A striking feature of loci differentially methylated between CVS and AC were the preferentially intermediate methylation levels of these loci in CVS in contrast to the extreme DNA-methylation levels in AC samples (Figure 2). DNA methylation in normal tissues shows for the majority of genes a bimodal distribution, i.e. full methylation or no methylation [20], [31]. This pattern was indeed observed in both AC and CVS samples. The enrichment of intermediate methylation levels in the genes differentially methylated between AC and CVS is inherent to this set of genes. A technical bias seems unlikely as the validity of the array has been proven previously and in the present set of samples by independent techniques. Also a bioinformatic bias seems unlikely due to the fact that intermediate methylation levels in CVS were present in 85% of the differentially methylated genes and in AC only in 15% of those genes. Moreover, the DNA methylation pattern of these CpG loci in AC was similar to the pattern found in peripheral blood of adults (data not shown). Finally, the genes hypermethylated in AC were significantly enriched for genes with LCPs (but depleted for HCP and PRC2 target genes in embryonic stem cells) suggesting a role in somatic tissue-specificity. Overall, these observations are in remarkable agreement with a recent report by Novakovic et al. [36] who showed that there is a progressive increase in average methylation in human placentas from first to third trimester. Using also the Infinium HumanMethylation27 BeadChip these authors have demonstrated that this progress particularly affects genes with an intermediate (beta value >0.6) methylation level and non-CpG island (i.e. LCP) genes. AC samples are usually obtained 2–10 weeks later in pregnancy than CVS samples. Therefore the intermediate methylation levels characteristic of CVS samples might reflect the different origin of the cells in such a sample. A different explanation is that in earlier weeks of pregnancy there is a higher epigenetic instability, comparable to the increased propensity for chromosomal mosaicism in the early placenta. The latter might be supported by the fact, that placental tissue also genetically is frequently mosaic [37].

Another feature which explains intra-sample heterogeneity might be contamination with maternal cells. By analysis of X-chromosomal methylation patterns we show (here) that such contamination might be variable and can even affect detection of the fetal sex via methylation-based X-inactivation patterns. In contrast to the CVS samples cultured cells derived from AC usually lack maternal contamination.

We failed to identify any association of maternal factors like maternal age, BMI or smoking status on the methylation pattern in AC and CVS likely due to the small size of the cohort for such analyses. In line with this, targeted analysis of the *F2RL3* locus recently shown to be differentially methylated in peripheral blood with regard to the smoking status [38] showed no different methylation levels in samples depending on the maternal smoking status.

Analyses of the association of karyotypic aberrations with DNA methylation levels had to be restricted to CVS samples due to the low number of AC samples with such changes. The intra-sample variability of CVS samples described above might be the cause that we only detected 15 loci, corresponding to 14 genes (Figures 4c and 4d) significantly differentially methylated between CVS samples with and without trisomy 18. In contrast, we identified a total of 464 loci (Figure 4; q<0.01) corresponding to 404 genes which were differentially methylated in samples with trisomy 21 as compared to CVS samples with a normal karyotype. It needs to be emphasized that the array applied in this study aims at a genome-wide coverage whereas several previous studies comparing DNA methylation between trisomic and normal CVS/placental samples focused on CpGs on the affected chromosomes, i.e. 18 or 21 [14], [23], [39]. A recent postnatal study investigated peripheral blood samples and isolated T-cells of individuals with Down syndrome as compared to controls on the same array platform like the one applied by us identified 108 genes (118 probes) and 140 CpGs (134 genes) to be differentially methylated in Down syndrome [40]. Nevertheless differences in the methylation profiles between individuals with and without trisomy 21 have been shown to be tissue specific [35].

Remarkably, in trisomy 21 fetuses studied herein more than 95% (387/404) of the differentially methylated genes were hypermethylated as compared to normal CVS samples. Only 3/387 hypermethylated (*COL6A2, H2BFS, RUNX1*) and 1/17 hypomethylated (*MCM3AP*) genes are located on chromosome 21. Thus, the trisomy 21 is associated with a genome-wide perturbance of DNA methylation not restricted to chromosome 21 that predominately leads to hypermethylation. This is in line with the pattern of differentially methylated genes observed by Kerkel et al. [40] on postnatal blood samples of individuals with trisomy 21. None of the nine genes identified by Kerkel et al. being aberrantly methylated in peripheral blood samples of patients with trisomy 21 have been identified by us. However, the focus of the study by Kerkel et al. and this study differs in the stage of development and the tissues analyzed. In our study, only 112 of the 404 genes belong to the 2014 genes showing tissue-specific differential methylation between CVS and AC suggesting that the deviation from the physiologic pattern in CVS might also be representative for other tissues. Remarkably, both differentially hypo- and hypermethylated genes in trisomy 21 were enriched for HCP content. Moreover, the 387 hypermethylated genes in trisomy 21 were significantly enriched for known PRC2 target genes in embryonic stem cells while genes with LCP were significantly depleted. This might suggest that many of those genes differentially methylated in trisomy 21 are indeed relevant for prenatal development. In line with this, the aberrantly methylated genes in cases with trisomy 21 show a remarkable enrichment of genes involved in processes of neuronal, heart, kidney, gut or facial bone (e.g. palate) development. Considering the phenotype of Down syndrome which frequently includes developmental disorders of the named organs it is intriguing to speculate that trisomy 21 exerts its pleiotropic effects via deregulation of an epigenetic modifier. Such a modifier might be localized in the Down syndrome critical region in chromosome 21 or could be deregulated indirectly e.g. through a transcript located on chromosome 21. Indeed, such a hypothesis could explain to a considerable extend the intra-individual variation in the Down syndrome phenotype as the levels of DNA methylation and its distribution in different tissues might vary and thus could explain differences in expressivity.

In summary, our extensive array-based survey of fetal sample obtained by routine prenatal invasive testing points to major tissue-specific differences of fetal DNA-methylation and gives rise to the hypothesis that part of the Down syndrome phenotype is epigenetically programmed in the first trimester of pregnancy.

## Supporting Information

Figure S1 **Reproducibility of the BeadArray based DNA methylation analyses as measured by Pearson coefficients.** Two CVS samples have been hybridized separately in independent experiments using different batches of arrays.
(TIF)

Figure S2 **Verification of results obtained from BeadArray analysis by bisulfite pyrosequencing (BPS).** 6 CpG loci have been analyzed both by BPS (A, upper table, values represent % methylation) and by BeadChip technology (A, lower table; average beta values are shown) in the same samples. The color code indicates methylation value (green: low, black: intermediate, red: high DNA methylation). (B) Scatter plots of results obtained in (A). Pearson’s product-moment correlation r^2^ = 0.69
(TIF)

Figure S3 **The majority of CpG loci present on the array are comparably methylated in CVS and AC samples.** Histograms showing the frequency of the DNA methylation values in CVS (A) and AC (B). Scatter plot comparing DNA methylation values of CVS and AC samples (C). Histograms and scatter plots show DNA methylation values of CpG loci not differentially methylated between AC and CVS (FDR>1×10^−15^, t-test). Pearson’s product-moment correlation r^2^ = 0.94.
(TIF)

Figure S4 **Hierarchic cluster analysis of DNA methylation values of 767 X-chromosomal CpG loci present on the array.** The upper panel on top of the heatmap indicates the sample type (green: AC, red: CVS) while the second panel indicates the fetuses’ sex (pink: female, cyan: male). The third panel indicates normal karyotype (yellow) versus Turner syndrome (black). Three CVS samples from male fetuses show a DNA methylation pattern similar to female samples (arrow). A blue bar at the right site indicates genes methylated specifically in male fetuses, an orange bar genes methylated in female samples and a black bar genes with tissue- specific DNA methylation.
(TIF)

Figure S5 **The AC sample derived from a pregnancy after ICSI showed a peculiar pattern of methylation.** PCA (A) and hierarchic cluster analysis of DNA methylation values of 2418 CpG loci differentially methylated between AC (green sphere (A) or green square (B)) and CVS (red sphere (A) or red square (B)) (t-test, FDR<1×10^−15^). One AC sample from ICSI is indicated by a blue sphere (A) or a blue square (B), respectively.
(TIF)

Table S1 **Clinical characteristics of the samples included in the study.**
(XLS)

Table S2 **Gene ontology terms (GO) significantly enriched in the group of genes differentially methylated between CVS and AC samples as compared to the entirety of loci present on the array (p<0.001, GOrilla tool: GOprocess).**
(XLS)

Table S3 **Gene ontology terms (GO) significantly enriched in the group of genes differentially methylated between CVS samples without chromosomal imbalances and samples with known trisomy 21 as compared to the entirety of loci present on the array (p<0.001, GOrilla tool: GOprocess).**
(XLS)

Table S4 **DNA methylation values (avg.beta and normalized data) of loci differentially aberrantly methylated in trisomy 21 (sheet 1) and trisomy 18 (sheet 2).** Data from CVS (trisomy and CVS) as well as from peripheral blood samples from normal female and male donors are included.
(XLS)
