# Supplementary figures and images for: DNA-Methylation Profiling of Fetal Tissues Reveals Marked Epigenetic Differences between Chorionic and Amniotic Samples
Source: PLoS One. 2012 Jun 19;7(6):e39014. doi: 10.1371/journal.pone.0039014 (PMC3378600; doi:10.1371/journal.pone.0039014)

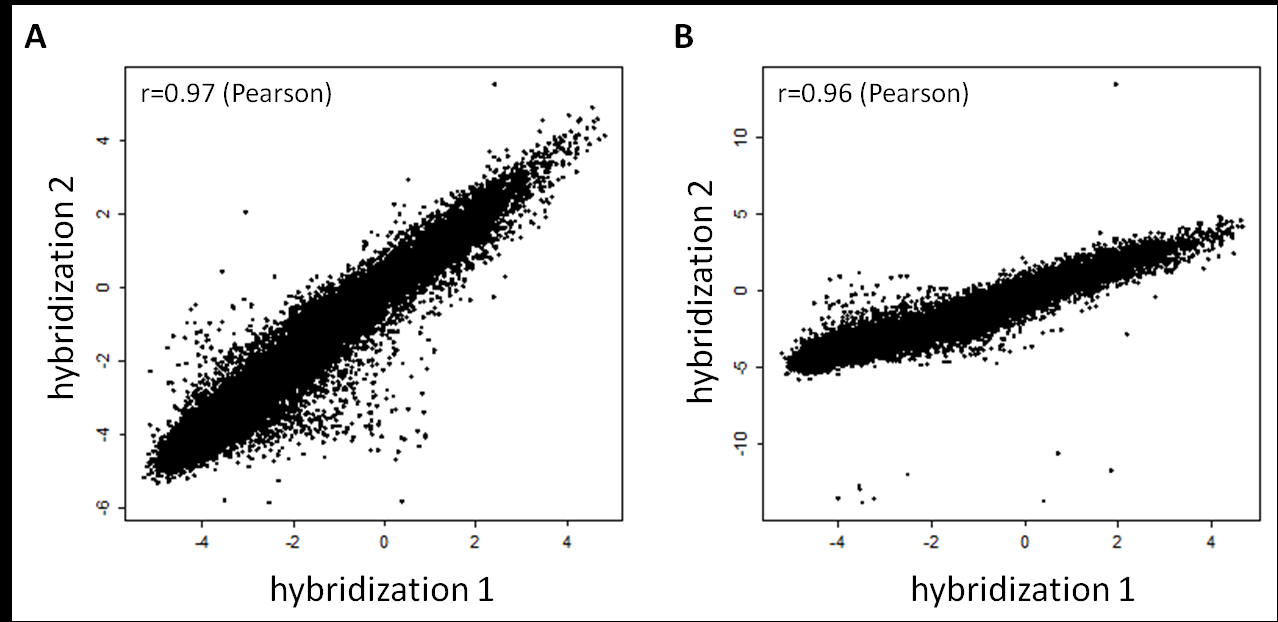

Supplement: Figure S1 — Reproducibility of the BeadArray based DNA methylation analyses as measured by Pearson coefficients. Two CVS samples have been hybridized separately in independent experiments using different batches of arrays. (TIF) [file pone.0039014.s001.tif]

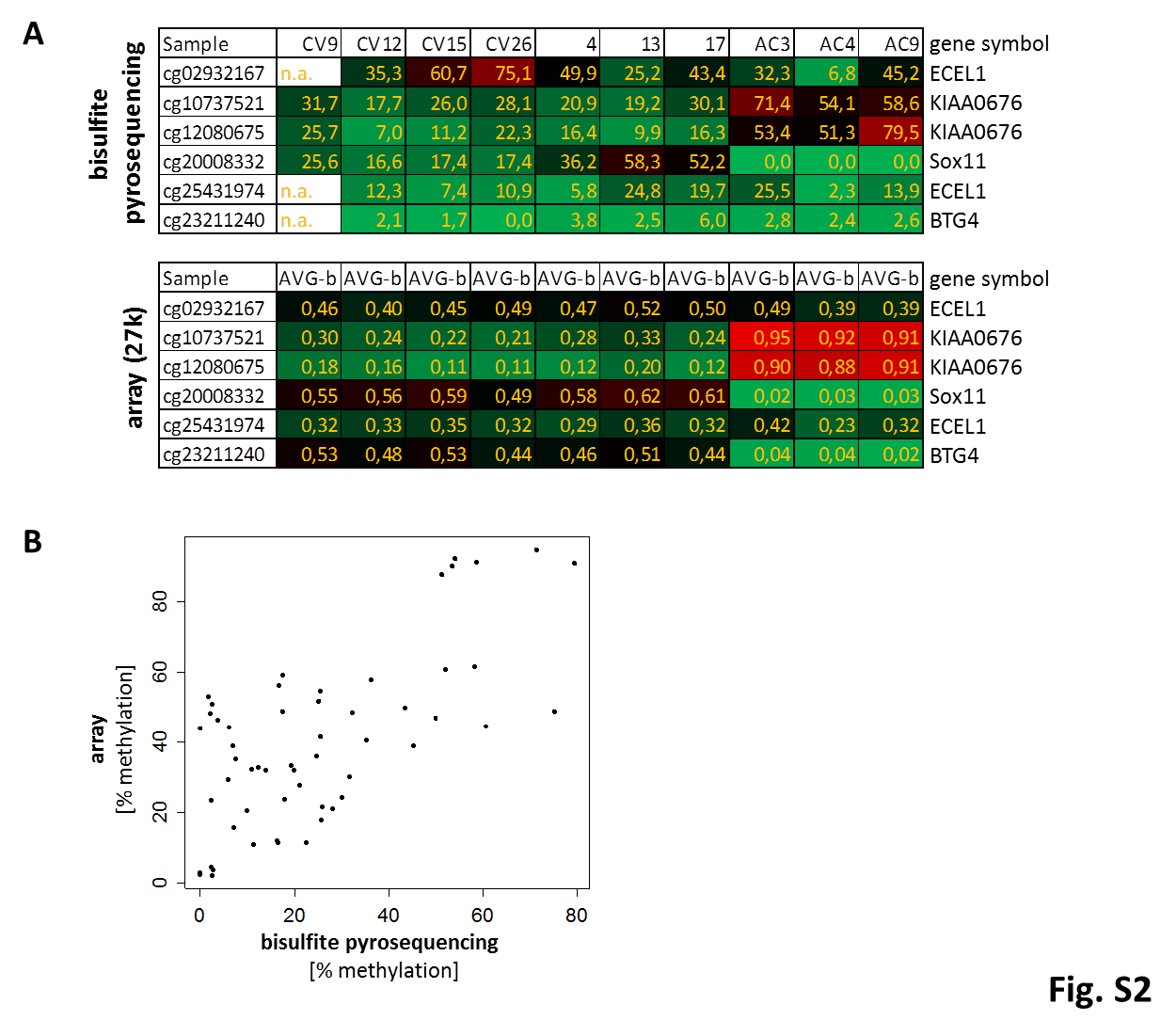

Supplement: Figure S2 — Verification of results obtained from BeadArray analysis by bisulfite pyrosequencing (BPS). 6 CpG loci have been analyzed both by BPS (A, upper table, values represent % methylation) and by BeadChip technology (A, lower table; average beta values are shown) in the same samples. The color code indicates methylation value (green: low, black: intermediate, red: high DNA methylation). (B) Scatter plots of results obtained in (A). Pearson’s product-moment correlation r2 = 0.69 (TIF) [file pone.0039014.s002.tif]

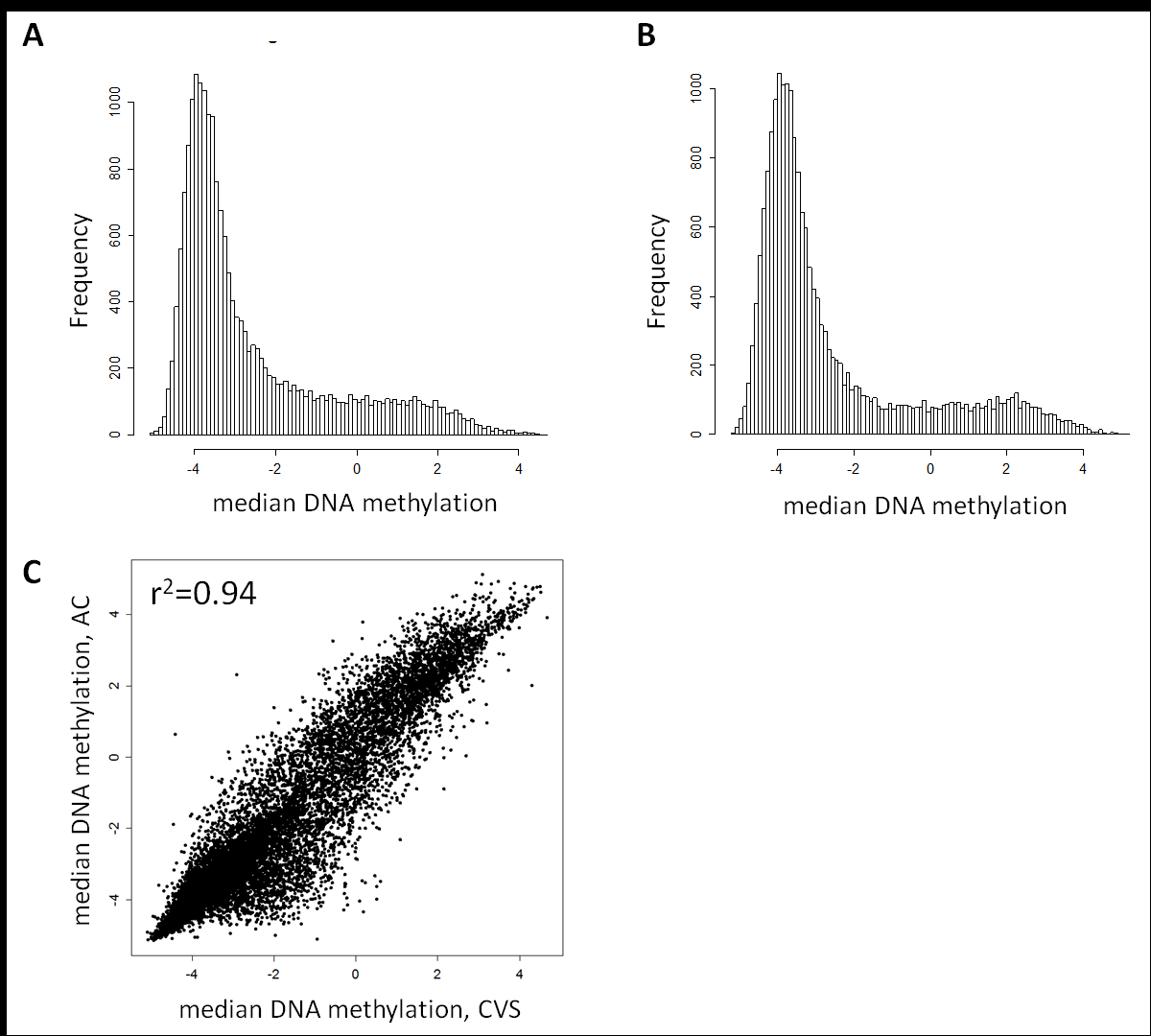

Supplement: Figure S3 — The majority of CpG loci present on the array are comparably methylated in CVS and AC samples. Histograms showing the frequency of the DNA methylation values in CVS (A) and AC (B). Scatter plot comparing DNA methylation values of CVS and AC samples (C). Histograms and scatter plots show DNA methylation values of CpG loci not differentially methylated between AC and CVS (FDR>1×10^−15^, t-test). Pearson’s product-moment correlation r2 = 0.94. (TIF) [file pone.0039014.s003.tif]

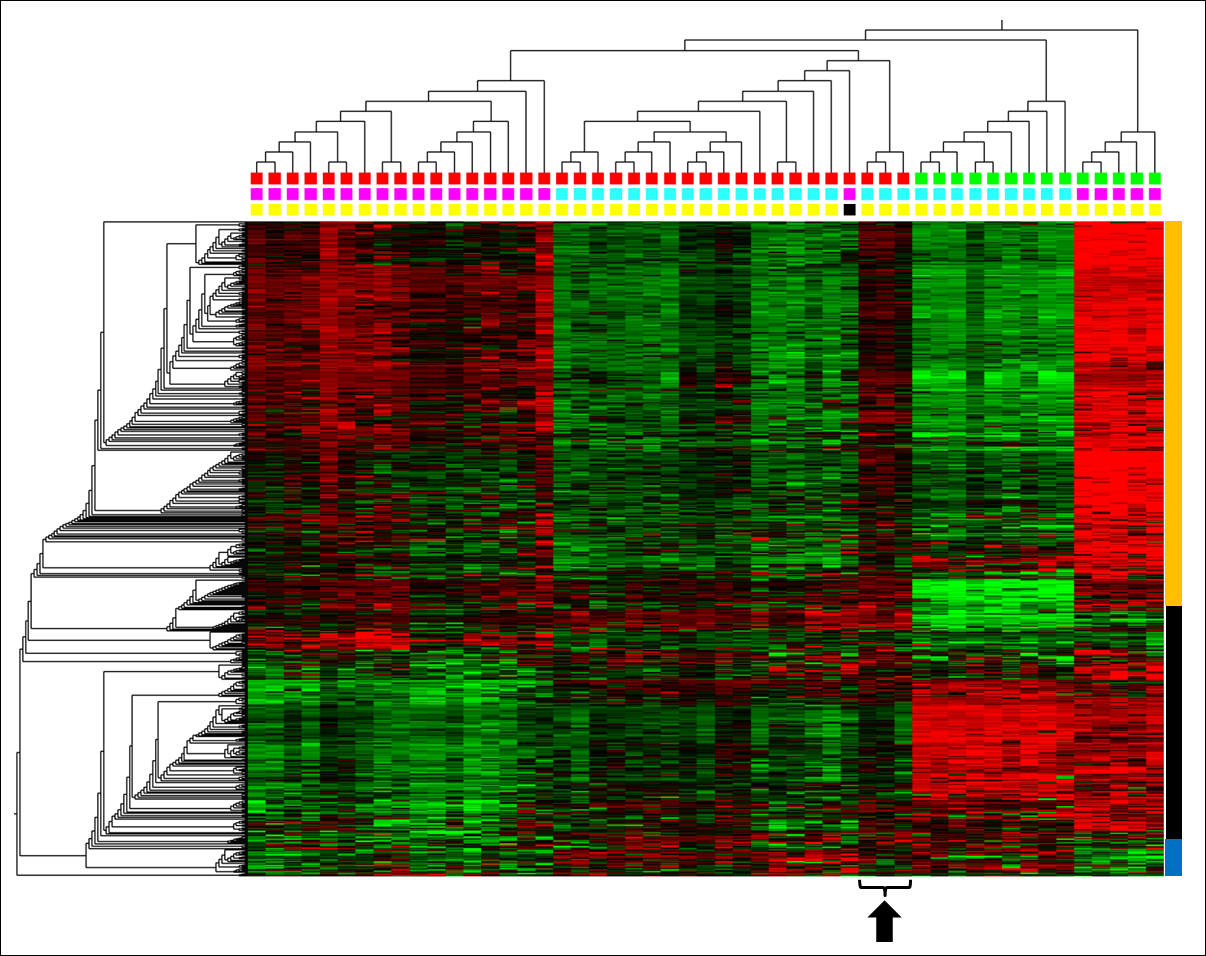

Supplement: Figure S4 — Hierarchic cluster analysis of DNA methylation values of 767 X-chromosomal CpG loci present on the array. The upper panel on top of the heatmap indicates the sample type (green: AC, red: CVS) while the second panel indicates the fetuses’ sex (pink: female, cyan: male). The third panel indicates normal karyotype (yellow) versus Turner syndrome (black). Three CVS samples from male fetuses show a DNA methylation pattern similar to female samples (arrow). A blue bar at the right site indicates genes methylated specifically in male fetuses, an orange bar genes methylated in female samples and a black bar genes with tissue- specific DNA methylation. (TIF) [file pone.0039014.s004.tif]

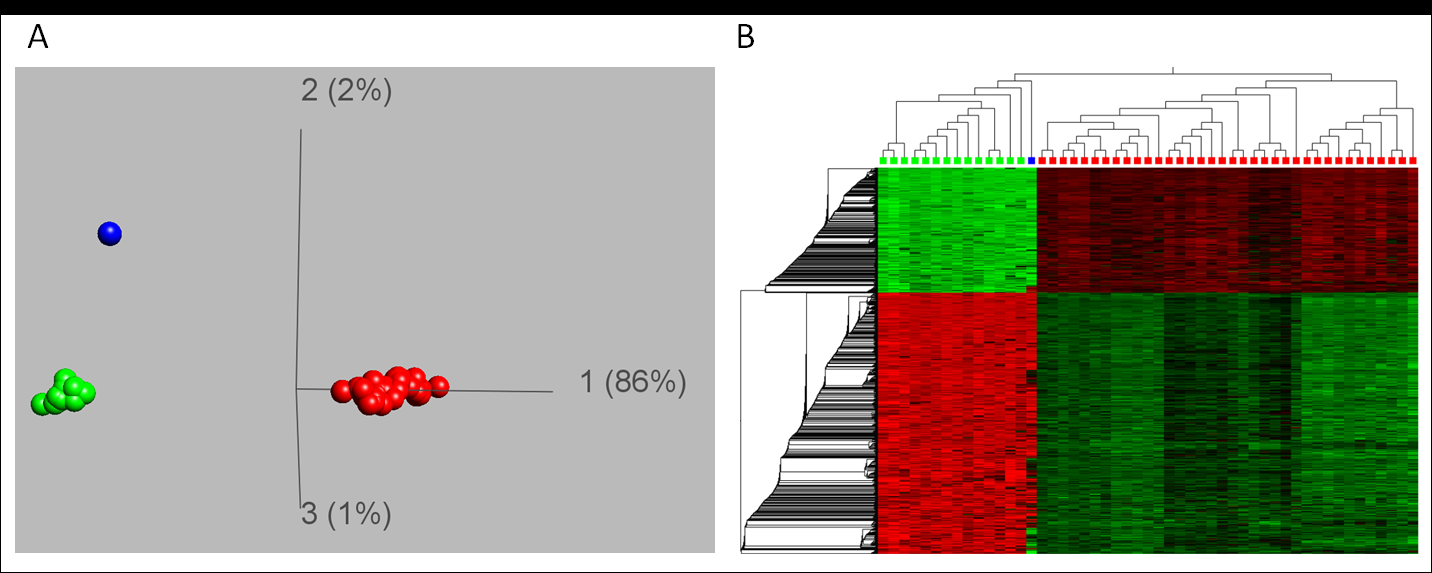

Supplement: Figure S5 — The AC sample derived from a pregnancy after ICSI showed a peculiar pattern of methylation. PCA (A) and hierarchic cluster analysis of DNA methylation values of 2418 CpG loci differentially methylated between AC (green sphere (A) or green square (B)) and CVS (red sphere (A) or red square (B)) (t-test, FDR<1×10^−15^). One AC sample from ICSI is indicated by a blue sphere (A) or a blue square (B), respectively. (TIF) [file pone.0039014.s005.tif]
